# Enhanced Impact Strength of Recycled PET/Glass Fiber Composites

**DOI:** 10.3390/polym13091471

**Published:** 2021-05-01

**Authors:** Marco Monti, Maria Teresa Scrivani, Irene Kociolek, Åge G. Larsen, Kjell Olafsen, Vito Lambertini

**Affiliations:** 1 Proplast, Via Roberto di Ferro 86, 15122 Alessandria, Italy; maria.scrivani@proplast.it (M.T.S.); irene.kociolek@proplast.it (I.K.); 2 SINTEF Materials and Nanotechnology, P.O. Box 124 Blindern, 0314 Oslo, Norway; age.larsen@sintef.no (Å.G.L.); Kjell.Olafsen@sintef.no (K.O.); 3 C.R.F. S.C.p.A. Group Materials Labs, C.so Settembrini 40, 10135 Torino, Italy; vitoguido.lambertini@crf.it

**Keywords:** poly(ethylene terephthalate), fiber reinforced composites, recycling, glass fibers, impact properties

## Abstract

In this paper, we report a study on the effects of different ethylene copolymers in improving the impact strength of a fiber-reinforced composite based on a recycled poly(ethylene terephthalate) (rPET) from post-consumer bottles. Different ethylene copolymers have been selected in order to evaluate the effects of the polar co-monomer chemical structure and content. The composite mixtures were prepared via melt extrusion, and the samples were manufactured by injection molding. Impact strength was evaluated using Izod tests, and a morphological study (FESEM) was performed. As a result, a composite with substantially improved impact properties was designed. This study demonstrates that a post-consumer PET from the municipal waste collection of plastic bottles can be successfully used as a matrix of high-performance, injection-molded composites, suitable for use in the automotive sector, among others, with no compromise in terms of mechanical requirements or thermal stability.

## 1. Introduction

Poly(ethylene terephthalate) (PET) is one of most used polymers for rigid packaging applications, and the only one whose technical performance puts it in the category of engineering polymers [1]. In the packaging industry in particular, PET is commonly used to make bottles and trays, which are typically manufactured via injection blow molding and thermoforming processes, respectively. As the packaging industry accounts for almost 40% of the global European plastic converters’ demand [2], there is a strong need for a highly efficient plastic recycling value chain.

Recycling is increasingly becoming the most promising approach to reducing the environmental impact of plastics, which is mainly related to end-of-life management [3]. For example, the “European Strategy for Plastics in a Circular Economy” [4], which was published in January 2018, established several targets to be reached, related to recycling. Indeed, by 2030, all plastic packaging in the EU market should be either reusable or recyclable in a cost-effective manner, and recycled plastics should become an increasingly valuable feedstock for industries. Moreover, Directive (EU) 2018/852 of the European Parliament [5] establishes minimum targets by weight for plastic recycling from packaging waste of 50% by the end of 2025, and 55% by the end of 2030. As a consequence, the challenge is two-fold: finding new applications for materials based on recycled plastics from post-consumer packaging, and at the same time improving their performance so that they comply with the technical requirements related to those applications. Effective recycling of post-consumer plastic packaging sourced from municipal waste collection is difficult, since it is typically constituted by a variety of different polymers and additives (such as pigments and chemical stabilizers) [6,7,8]. At present, only a fraction of the total amount of collected plastic packaging is actually recycled, and only a part of this fraction is efficiently recycled into high-value products [9]. 

Today, PET bottles represent an excellent example of efficient recycling, as the recycled flakes commercially available reach extremely high levels of purity, and can be used even in food packaging applications [10,11,12]. However, multicolored PET bottles from municipal collection systems, which are separately collected in Italy by the national collection system, cannot be used again in the food industry to manufacture bottles and trays. This limitation makes this material feedstock highly available for many technical applications. In particular, it can be used as a matrix of injection-molded, fiber-reinforced composites, especially in the automotive sector, which is typically looking for cost-effective “green” solutions [13,14]. The technical performance of PET, especially in terms of thermal stability and thermo-mechanical resistance, allow one to use it as a potential replacement for polyamides (PA) for those applications in which polyolefins typically cannot be used due to their poor thermal resistance [15]. Several studies have reported on the manufacturing of injection-molded, PET-based, fiber-reinforced composites [15,16,17,18]. For example, Alqaflah et al. [16] prepared glass-fiber composites based on blends of PET and LLDPE, demonstrating the positive contribution of LLDPE in improving impact strength. Arencón et al. [17] studied the thermal and mechanical properties of short glass-fiber-reinforced PET, focusing on the influence of three of the variables involved in the injection-molding process: mold temperature, holding pressure time, and closed mold time. As a result, both higher mold temperatures and longer closed mold times led to the highest values of developed crystallinity, with beneficial effects on both mechanical properties and thermal resistance. The possibility of using recycled PET as a matrix for the preparation of fiber-reinforced composites has been also widely investigated [13,19,20,21], as its properties may be significantly reduced when compared with its virgin counterpart [22]. For example, Giraldi et al. [19] studied the thermal and mechanical properties of recycled PET reinforced with glass-fiber recycled poly(ethylene terephthalate) composites, focusing on the influence of screw speed and torque produced during the extrusion process. Mondadori et al. [20] developed composites based on recycled PET and short glass fibers (GFs) in different compositions, using recycled bottle-grade PET either in its degraded form, or in a solid-state polymerized (SSP) form. The experimental elastic modulus values of PET/GF composites were successfully compared with theoretical ones obtained using the Halpin–Tsai model. As a result, slightly better mechanical strength was achieved for the composites based on the SSP recycled PET, which is most likely due to its highly entangled amorphous phase, arising from its higher molecular weight. 

Despite the good mechanical and thermal properties of PET composites, they have a limited impact strength, which hinders their use in several applications. Many studies have shown that the addition of different copolymers can strongly improve the impact strength of virgin and recycled PET [23,24,25,26]. For example, Bocz et al. [23] studied the effect of PET’s molecular weight on the efficiency of the reactive toughening of ethylene–butyl acrylate–glycidyl methacrylate (EBA–GMA) copolymers. Kunimune et al. [26] studied the effect of the content of ethylene–glycidyl methacrylate (E–GMA) copolymers and their processing condition on the morphology and mechanical performance of recycled PET.

This paper reports a study on improving the impact strength of a glass-fiber-reinforced composite (GFRC) based on recycled PET sourced from post-consumer bottles from municipal waste. Our ultimate goal is to develop a highly crystalline PET composite suitable for use via injection molding, and with mechanical properties representing the best compromise in terms of stiffness, impact strength, and thermo-mechanical resistance. In particular, this study is focused on the effect of the chemical composition of the polar moiety of different ethylene copolymers. The co-monomers were either methyl acrylate or methacrylic acid partially neutralized with sodium ions. In the case of methyl acrylate, the effects of different amounts and of the presence of a reactive co-monomer, which is able to graft to COOH end-groups of the PET chain, has been investigated.

## 2. Materials and Methods

### 2.1. Materials

**Figure 1:**
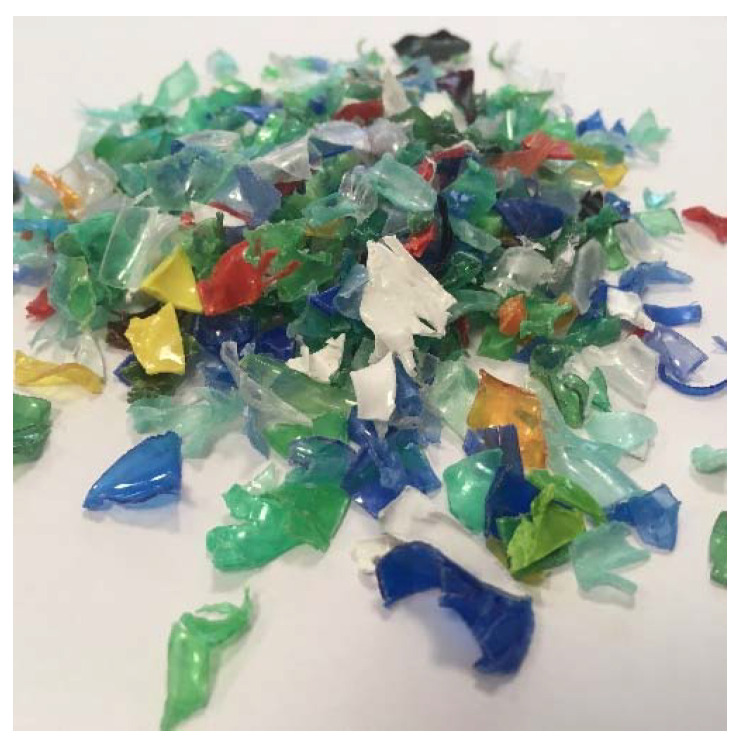
PET flakes, as received from the recycler.

Recycled PET (trade name: PETALO MC300/1) was supplied by Dentis Recycling Italy s.r.l. (Cuneo, Italy). According to the producer, this was a post-consumer grade sourced from multicolored PET bottles (from the municipal collection system), with a contamination level of less than 60 ppm (PVC, polyolefine, metals, and others). The real PET flake density was 1.35–1.38 g/cm^3^, and the apparent PET flake density was 0.65–0.70 g/cm^3^. The claimed intrinsic viscosity was a minimum of 0.7 dL/g. The dimensions of the flakes were in the range of 0.6–8 mm. Figure 1 shows a picture of the PET flakes, as they were received. Chopped strands of E-Glass fibers (CS 7967 (26/1493) D) with sizing specifically suitable for PET were purchased from Lanxess (Cologne, Germany). As claimed by the producer, these have a diameter of 10 µm, and an average length of 4.5 mm. The size content was approximately 0.90 wt%. Two different processing aids were used in order to make the final composites suitable for conversion via injection molding: one as a nucleating agent (Bruggolen P252); and the other as a lubricating agent (Bruggolen P130). Both were purchased from Brüggemann GmbH (Heilbronn, Germany).

Four different copolymers were used to improve the impact properties and the homogeneity of the mixtures, namely: Elvaloy AC12024S by Dow (Schkopau Germany), LOTRYL 29MA03 by SK global chemical (Seoul, Korea), LOTADER AX8900 by SK global chemical (Seoul, Korea), and Surlyn 8940 by Dow (Schkopau Germany). A detailed description of these additives is reported in Table 1. The effect of different amounts of polar methyl acrylate moieties on the rubber particles’ sizes was evaluated by the introduction of E-MA/24 and E-MA/29. The selection of E-MA/24-GMA copolymers allowed us to highlight the improvement that the reactive epoxy group has on matrix–rubber compatibility. This polymer, in fact, showed the ability to graft directly with the COOH end-groups of the polymer matrix during extrusion, assuring better interconnection of the polymers. Finally, the effect of a free acid moiety was studied using an EMAA ionomer. Such a structure may have a great affinity for PET, having similar acid functionality, and it may react with the PET’s OH end-group.

### 2.2. Processing

**Figure 2:**
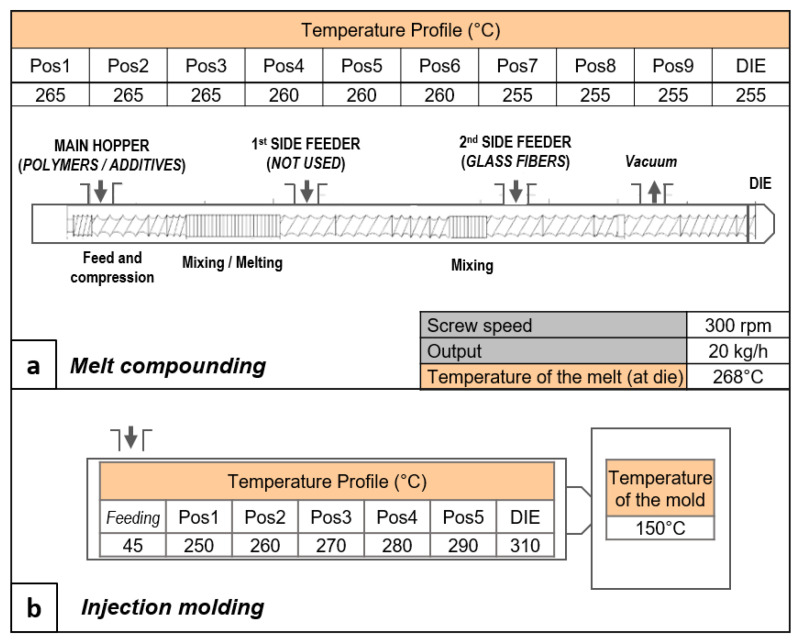
Scheme of the selected screw profile for the melt extrusion process (dye on the left side).

All of the additives were homogeneously mixed with PET in a Leistritz 27E co-rotating twin-screw extruder. The screws had a diameter (D) of 27 mm and a length-to-diameter ratio (L/D) of 40. The processing parameters—i.e., screw speed, output, temperature, and screw profiles—are reported in Figure 2a. The screw profile was chosen as a compromise between the dispersion of the elastomers and glass fibers, and the degradation of the PET due to the process shear stress. The PET flakes were dried before melt compounding (150 °C for 6 h), with a Moretto dryer (Padova, Italy), model XD21, with a resulting humidity of 40 ppm (measured using Karl Fischer titration, data not reported in this paper). The PET flakes were kept in a nitrogen atmosphere in the dosing unit in order to avoid humidity reabsorption. Table 2 reports the recipes of the produced mixtures and the code names that will be used hereinafter.

The produced materials were injection molded for the production of the specimens for characterization. The injection molding was carried out using an Arburg AllRounder 370S 500/170 injection molding press (Loßburg, Germany) (Clamping force: 500 kN; Max. swept volume: 58 cm^3^; Max. Injection Pressure: 2500 bar). The mold temperature was set at 150 °C in order to promote the PET’s crystallization, with the ultimate goal of maintaining the highest thermo-mechanical resistance. The selected temperature profile is reported in Figure 2b. The pellets were dried before the injection process, following the same procedure described for melt compounding.

### 2.3. Characterization

Several characterization tests were performed on the materials. 

The intrinsic viscosity (IV) was calculated using the Billmeyer relationship η = 0.25 (η_r_ − 1 + 3lnη_r_)/C, (with η_r_ = relative viscosity (t/t_0_) and C polymer solution concentration, g/dL). The polymer was dissolved in a phenol/1,1,2,2-tetrachloroethane 60:40% (*w*/*w*) mixture, then filtered using a Gooch filter funnel (pore size 100–160 µm) in order to remove glass fibers and rubber. The solution concentration was corrected in order to maintain the correct polymer concentration, as described in ASTM D4603.

Tensile tests were performed according to ISO 527 standards, using a Z010 dynamometer (Zwick Roell, Ulm, Germany) with a 10 kN load cell (1 mm/min Elastic modulus speed–50 mm/min test speed). The test temperature was set at 23 °C, with 50% relative humidity (RH). The results are reported in terms of elastic modulus, stress at yield and break, and elongation at yield and break. 

Izod impact tests were performed in both the notched (type-A) and un-notched configurations, according to ISO 180 standards, using an ATS FAAR IMPats-15 impact tester with a pendulum impact energy of 1 J and a velocity of impact of 3.46 m/s. The A-type notch was obtained according to ISO2818, using a 6816 Notchvis Instron-CEAST (Norwood, MA, USA) device. The test temperature was set at 23 °C (50% RH). The results are reported using the impact strength value, defined as the impact energy absorbed while breaking a sample, referred to the original cross-sectional area, and the type of failure (according to ISO180 standard: C = complete break (including hinge break H); P = partial break; N = non-break). 

The morphological study on the prepared samples was performed using an FEI Nova nano SEM 650 (FEI Company Hillsboro, OR, USA) scanning electron microscope (SEM). The studied samples were the fracture surfaces obtained after impact testing. The surfaces were coated with a thin layer of evaporated gold in order to make them conductive. The evaluation of the copolymer particle size was performed using LAS 3.6 software. About 600 measurements for each material were taken.

Differential scanning calorimetry (DSC) was performed on a representative specimen cut from the whole cross-sectional area of the injection-molded samples. A Q800 DMA, from TA Instruments (New Castle, DE, USA), was used. A single heating scan was set from 25 to 300 °C, with a heating rate of 10 °C/min. The degree of crystallinity was calculated according to the equation:

(1) $$X_{c}=\frac{100}{100-wt\%}\frac{\Delta H_{m}}{\Delta H_{0}}$$

where *X_c_* is the crystalline fraction of the matrix, wt% is the total content of additives (i.e., glass fibers, processing aids and copolymers), and ΔH_0_ is the theoretical crystallization enthalpy of 100% crystalline PET, whose value is 140 J/g according to [27].

## 3. Results and Discussion

It is well known that PET is highly sensitive to hydrolytic degradation [1]. For this reason, humidity control throughout all of the processing phases is crucial. The intrinsic viscosity values represent an indirect approach to estimating the molecular weight of the polymer, and have been used to understand the potential degradation induced either by the processing or by the interaction with the added copolymers. Table 3 reports the obtained results for all of the produced materials. As can be seen, all of the materials apart from PET-4 show an IV value slightly above 0.6. This means that a slight hydrolytic degradation has occurred, since the raw material has an IV above 0.7 (reduction of about 15%). It should be noted that the manufacturing process of the injection-molded sample is based on two subsequent melting of PET, the former for mixing the additives; the latter for injection molding, doubling the possibility of facing hydrolytic degradation. Taking into account this aspect, and the repeatability in all the mentioned cases (robustness of the process layout), together with the relatively low value of this reduction, it can be concluded that there is an acceptable quality level of the produced sample. On the other hand, PET-4 shows a significant IV reduction, which cannot be attributed to the processing condition. This reduction can be attributed to the un-neutralized methacrylic acid moieties, which are able to act as chain scission catalyst, inducing the production of acetaldehyde. At the same time EMAA is more prone to absorbing moisture than the other impact modifiers tested, which may further increase the degradation of PET chains.

Table 4 reports the results of the tensile tests. As can be seen, although a brittle-to-ductile transition did not fully occur, all of the used copolymers led to a slight reduction in stiffness, with a reduction of the elastic modulus in the range of 10%, and an increase in ductility, represented by a higher strain at break. This is the expected effect of embedding elastomers in the PET matrix. The addition of the reactive copolymer (E-MA/24-GMA, PET-3) led to the highest reduction in stiffness, which can be attributed to chemical interaction between the PET chain and the glycidyl methacrylate group. On the other hand, a slight increase in ductility can be attributed to the increase in the methyl acrylate weight fraction as a consequence of the higher fraction of the polar moiety of the copolymer that is able to interact deeply with PET chains. 

Table 5 shows the results of the impact tests, both in the notched and un-notched configurations. As can be seen, the presence of the 2 mm deep A-type notch drastically reduces the impact strength. A similar reduction in the presence of a 2 mm-deep notch has been reported by Ogazi-Onyemaechi et al. [28], who studied the effects of V-notches on the fracture behavior of injection-molded PET. 

As for the notched configuration, E-MA/24, E-MA/29, and E-MA/24-GMA have similarly strong effects on impact strength, with slightly better performance when using the reactive copolymer (E-MA/24-GMA, PET-3), and no significant difference related to the weight fraction of methyl acrylate. On the other hand, E-MAA (PET-4) does not seem to have a significant effect. As for the un-notched configuration, the reactive copolymer (E-MA/24-GMA, PET-3) show the greatest effect on impact strength, further demonstrating that the glycidyl methacrylate group allows for the formation of a stable dispersed phase by grafting the elastomeric copolymer to the PET chains and simultaneously reducing the interfacial tension. The unreactive copolymers (E-MA/24, PET-1, and E-MA/29, PET-2) show only a limited toughening effect. EMAA has only a limited effect, even in the un-notched configuration.

An in-depth analysis of the impact results allows one to observe that the effects of the used copolymers are more pronounced in the notched configuration, when compared with the reference (PET-0), as reported in Table 5. Furthermore, both the unreactive (E-MA/24 and /29) and reactive (E-MA/24-GMA) copolymers lead to an increase in impact strength greater than 50% with respect to PET-0 in the notched configuration, while only a limited effect is produced by unreactive (E-MA) copolymers in the un-notched configuration, with the impact strength value increasing with increasing methyl acrylate content, from 24% to 29%. As reported in Table 5, the reactive copolymer leads in both cases to an impact strength increase of about 55%. This means that, when chemically bonded with the PET chains, E-MA/24-GMA is able to hinder both the crack initiation and the crack propagation. On the other hand, the effects of both of the unreactive copolymers are significantly higher in the notched configuration, and this can be explained by the fact that the weak interaction between E-MA particles and the continuous PET matrix leads to a poor contribution of E-MA particles in hindering the crack initiation, which begins on the external layer of the sample, constituted by PET. The notch presence allows E-MA particles to come out to the outer surface and to act as toughening agents by hindering the crack propagation.

**Figure 3:**
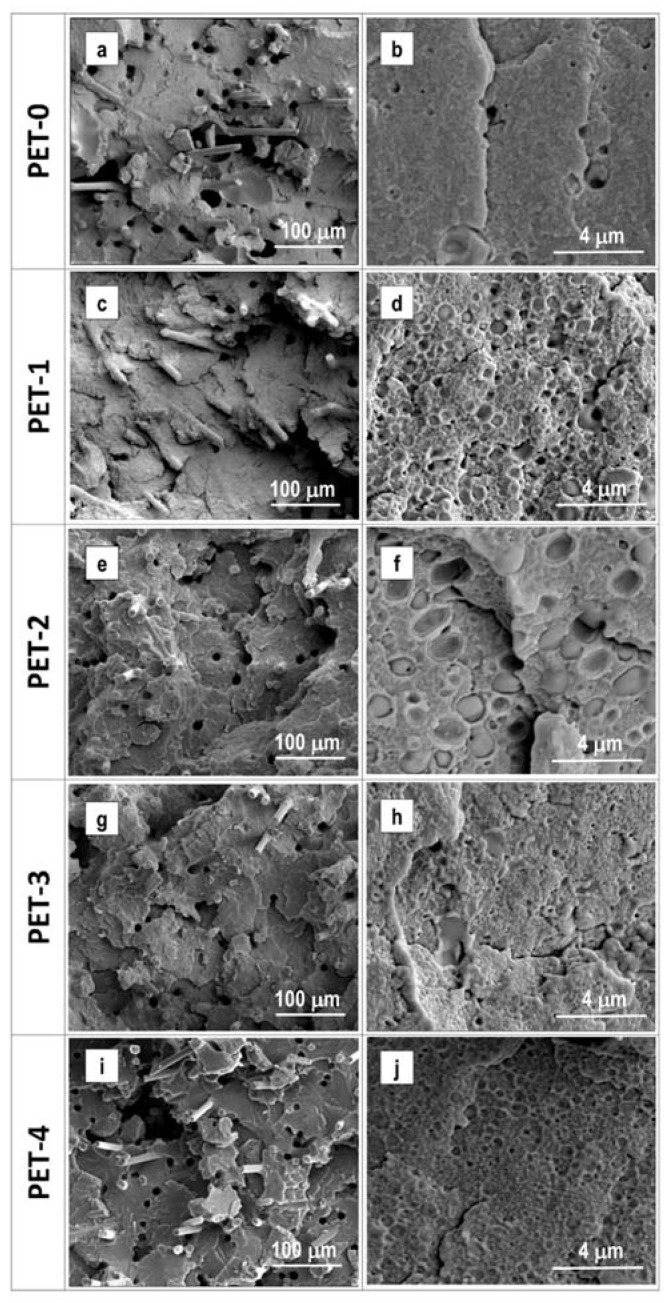
SEM micrographs of the produced materials. (**a**,**b**) PET-0; (**c**,**d**) PET-1; (**e**,**f**) PET-2; (**g**,**h**) PET-3; (**i**,**j**) PET-4. Left column reports the micrographs taked at 300X, right column at 10,000X.

In order to support the interpretation of the mechanical results, a morphological study has been performed. Figure 3 shows SEM micrographs of the produced materials at different enlargements, which are particularly useful for focusing on different morphological aspects. At the lowest enlargement (300×), all of the materials show small holes, whose dimensions are in the range of 10 microns, and seem not to vary with the observed material. This can most likely be attributed to the formation of acetaldehyde as a product of degradation, which typically appears in PET-based, injection-molded components. The micrographs obtained at the highest enlargement (10,000×) allow for the monitoring of the rubber particles’ sizes and their potential interfacial bonding with the PET chain. An in-depth analysis of all of the obtained micrographs allows us to calculate the mean dimensions of the rubber particles in all of the materials. The data are reported in Table 6. As can be seen, the non-reactive copolymers (E-MA/24 and E-MA/29) tend to form the largest rubber particles.

SEM images show clearly that non-reactive copolymers have weak interfacial bonding with PET. Many rubber particles jumped out of the fractured sample, which is evidence that these particles are chemically separated from the PET matrix. The introduction of the reactive functionality (GMA group) enables reduction of the rubber particles’ sizes. Observing the PET-3 sample, it is evident that the E-MA/24-GMA particles have strong interfacial bonding with the PET matrix, which is induced by the grafting of the glycidyl methacrylate group to the COOH end-groups of the PET chains. The obtained results are coherent with what was reported in previous literature [25,29,30,31,32]. For example, Zong et al. [25] showed that the reactive copolymers result in particles whose size is smaller than that of those obtained from the unreactive polyethylene octane elastomer, due to their deeper interaction with PET. Martin et al. [29] showed that polybutylene terephthalate (PBT) melt mixed with an ethylene–ethyl acrylate copolymer (E–EA) has greater particle size than an ethylene–methyl acrylate–glycidyl methacrylate copolymer (E–MA–GMA). Moreover, the E–MA–GMA also showed a much smoother surface, thanks to its chemical interaction with the PBT matrix. Cheng et al. [30] also showed that an average dimension below 500 nm can be successfully obtained with the reactive copolymers.

Dompas et al. [31] developed a model that predicts a critical minimum particle size for cavitation: below this threshold, which is related to the polymer matrix of the continuous phase, particles cannot cavitate, and their toughening efficiency decreases. Gloaguen et al. [32] studied rubber-toughened poly(methyl methacrylate), and demonstrated that the toughening effect becomes relevant only when the particle size is higher than 300 nm. This is in agreement with the results obtained with EMAA, whose domains in the system are around 300 nm due to their strong polar interaction with PET.

**Figure 4:**
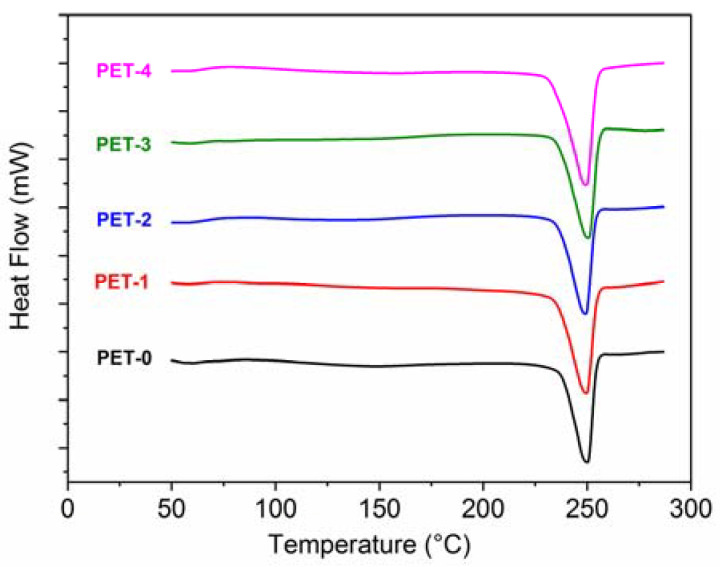
DSC curves (first heating scan) of the injection-molded samples.

Figure 4 and Table 7 report the results of the DSC analysis. Figure 4 shows the thermograms of the first heating performed on the injection-molded sample.

Since PET is a semi-crystalline polymer with a slow crystallization rate, its typical heating curve may show the presence of an exothermic peak caused by the cold crystallization of the amorphous phase, as reported for instance by Kunimune et al. [26]. The absence of a cold crystallization peak in the curve of Figure 4 suggests that the maximum level of crystallinity has been reached during injection molding, further confirming the obtained optimization of the processing condition, with no significant difference among the studied materials.

As for the shape of the melting peak, it has been reported [22] that in the absence of nucleating agents, PET chains may crystallize in different lamellar shapes: this is reflected in a broad melting curve having multiple melting points. As it is possible to observe from Figure 4, a single and narrow melting peak has been obtained with all of the materials. This is most likely related to a homogeneous spherulitic growth of the crystals, favored by the presence of different additives.

Table 8 reports the results of the heat deflection temperature tests. As can be seen, the HDT values are in all cases typically high for PET formulations, which may be due to the occurred crystallization, further confirming the obtained optimization of the processing condition. The presence of the copolymers, while reducing the stiffness, tends to lower the HDT value. In particular, this effect is more pronounced for PET-3 and PET-4. PET-3 is the material with the lowest elastic modulus and the highest impact properties: this may be related to its lower load-bearing capacity under increased thermal heating. PET-4 has a higher fraction of amorphous phase, and this can be attributed as a cause of the lower deflection temperature.

## 4. Conclusions

In this paper, we studied the influence of several ethylene copolymers on the impact strength of a fiber-reinforced composite based on PET recycled from post-consumer bottles. The composite mixtures were prepared via melt extrusion and the samples were manufactured via injection molding. A decrease in molecular weight was produced by the process, due to the effect of residual moisture. Nevertheless, good mechanical properties were obtained, demonstrating that the reduction in molecular weight is acceptable to produce a fiber-reinforced injection molding grade. The impact properties were studied using Izod tests, and a morphological study (FESEM) was performed. 

The copolymer functionalized with the glycidyl methacrylate (GMA) group (E-MA/24-GMA) allows for the greatest improvement of the impact properties. This is due to the capacity of GMA to react with the COOH end-groups of PET chains and create a stable dispersed phase, thus reducing the interfacial tension. As for the content of methyl acrylate (MA), the obtained results seem to show a slight improvement in impact properties as a result of increasing the MA content. Eventually, the methacrylic acid does not show any significant improvement in impact properties, and this can be attributed to the higher level of degradation that it induces to PET.

The absence of a cold crystallization peak in the DSC curves suggests that the maximum level of crystallinity has been reached during injection molding, further confirming the obtained optimization of the processing condition.

This study has demonstrated that a post-consumer PET sourced from the municipal waste collection of plastic bottles can be successfully used as a matrix of high-performance injection-molded composites, suitable for use in many technical applications as a replacement for other engineering polymers, such as polyamides and polybutylene terephthalate, with no compromise in terms of mechanical requirements or thermal stability.

## Figures and Tables

**Table 1:** Description of the additives used for impact modification.

Chemical Nature		Trade Name	Chemical Structure
E-MA/24	Copolymer of ethylene and methyl acrylate (24 wt%)	Elvaloy AC12024S	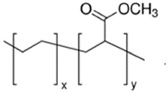 *y = 24% mole*
E-MA/29	Copolymer of ethylene and methyl acrylate (29 wt%)	LOTRYL 29MA03	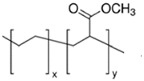 *y = 29% mole*
E-MA/24-GMA	Random terpolymer of ethylene, methyl acrylate (24 wt%), and glycidyl methacrylate (8%)	LOTADER AX8900	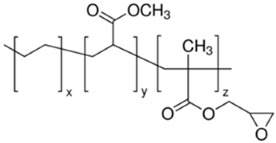
E-MAA	Copolymer of ethylene and methacrylic acid, partially neutralized with Na+	Surlyn 8940	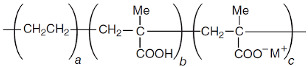 *M^+^ = Na^+^*

**Table 2:** Produced recipes and their related code names.

Ingredient	PET-0	PET-1	PET-2	PET-3	PET-4
r-PET	78.5	73.5	73.5	73.5	73.5
Lubricant agent	1	1	1	1	1
Nucleating agent	0.5	0.5	0.5	0.5	0.5
Glass fibers	20	20	20	20	20
E-MA/24		5			
E-MA/29			5		
E-MA/24-GMA				5	
E-MAA					5

**Table 3:** Results of intrinsic viscosity.

Material	IV (dL/g)
PET-0	0.610
PET-1	0.608
PET-2	0.602
PET-3	0.619
PET-4	0.558

**Table 4:** Results of the tensile tests.

Material	Elastic Modulus (Mpa)		Yield Stress (Mpa)		Strain at Yield Stress (%)		Stress at Break (Mpa)		Strain at Break (%)	
	*Avg*	*StDev*	*Avg*	*StDev*	*Avg*	*StDev*	*Avg*	*StDev*	*Avg*	*StDev*
PET-0	7870	(60)	120	(2)	2.0	(0.1)	119	(1)	2.0	(0.1)
PET-1	7174	(37)	107	(1)	2.1	(0.1)	104	(3)	2.3	(0.1)
PET-2	7144	(97)	104	(1)	2.2	(0.1)	99	(2)	2.6	(0.1)
PET-3	6896	(98)	102	(1)	2.2	(0.1)	100	(2)	2.5	(0.1)
PET-4	7124	(59)	106	(1)	2.2	(0.1)	106	(1)	2.2	(0.1)

**Table 5:** Results of the Izod impact tests. All of the samples show a C-type failure mode.

Material	Notch Type	IZOD Impact Strength, Notched (23 °C) (KJ/M2)		Impact Strength Increase with Respect to PET-0 (%)	Notch Type	IZOD Impact Strength, Unnotched (23 °C) (KJ/M2)		Impact Strength Increase with Respect to PET-0 (%)
		*Avg*	*StDev*			*Avg*	*StDev*	
PET-0	A	5.2	1.4		-	26.4	1.8	
PET-1	A	8.1	0.8	56%	-	29.6	1.8	12%
PET-2	A	8.1	1.1	55%	-	32.5	3.2	23%
PET-3	A	8.4	0.7	60%	-	40.3	4.2	52%
PET-4	A	5.6	1.3	7%	-	28.8	2.0	9%

**Table 6:** Measured rubber particle size.

Material	Rubber Particle Size (nm)	
	*Avg*	*StDev*
PET-1	550	245
PET-2	639	264
PET-3	404	234
PET-4	329	110

**Table 7:** Results of the DSC tests.

Material	Tm (°C)	Xc (%)
PET-0	250.3 ± 0.1	23.2 ± 0.9
PET-1	250.7 ± 1.4	23.2 ± 0.3
PET-2	250.4 ± 1.0	22.4 ± 1.4
PET-3	250.7 ± 0.2	23.4 ± 1.0
PET-4	248.8 ± 0.7	17.2 ± 0.6

**Table 8:** Results of the HDT Tests.

Material	*HDT (°C) @ 1,82 MPa*	
	*Avg*	*StDev*
PET-0	184	7
PET-1	186	6
PET-2	175	5
PET-3	169	4
PET-4	171	5

## Data Availability

The data presented in this study are available on request from the corresponding author(s).
